# Association between atmospheric SO_2_ exposure and severity of respiratory infections in infants: a systematic review

**DOI:** 10.1590/S1678-9946202668045

**Published:** 2026-07-20

**Authors:** Sandra Elisabete Vieira, Alexandre Archanjo Ferraro

**Affiliations:** 1 Universidade de São Paulo, Faculdade de Medicina, Departamento de Pediatria, São Paulo, São Paulo, Brazil

**Keywords:** SO_2_, Air pollution, Infants, Respiratory infections, Bronchiolitis, Pneumonia

## Abstract

Environmental factors may influence the severity of lower respiratory infections, a major cause of morbidity and mortality in infants. Sulfur dioxide (SO_2_) has biologically plausible pro-inflammatory and epithelial effects. This PRISMA 2020–compliant systematic review synthesized observational studies published between 2015 and 2025, selected to ensure methodological comparability and contemporary relevance, examining the association between atmospheric SO_2_ exposure and severity-related respiratory outcomes in infants. Thirteen studies met eligibility criteria. Most evaluated short-term exposure using daily lag structures (lag01 to lag03) or 1–2 week averages, whereas one birth cohort assessed annual exposure during the first year of life. All studies adjusted for meteorological variables, and most incorporated multipollutant regression models. Owing to substantial heterogeneity in exposure metrics, increments, lag structures, and effect scales, a meta-analysis was not performed. Across studies, short-term SO_2_ exposure was most consistently associated with increased hospitalization for ALRIs, longer length of stay, and progression to severe disease. Effect estimates per 10 µg/m^3^ were modest but statistically significant in specific lag windows. In one cohort, higher annual exposure was associated with severe ALRI (IRR 2.81; 95% CI 1.35–5.83), and one hospital-based study reported increased mortality (OR 2.04; 95% CI 1.33–3.13). Associations were less consistent in low-exposure settings or in models with strong multipollutant collinearity. Overall, the evidence suggests context-dependent associations between short-term SO_2_ exposure and increased severity of respiratory infections in infants, particularly hospitalization, longer length of stay, and progression to severe disease. Given the observational and heterogeneous nature of the data, findings should be interpreted cautiously.

## INTRODUCTION

Acute lower respiratory infections (ALRI), particularly bronchiolitis and pneumonia, remain the leading causes of morbidity and mortality in infants worldwide^1-5^. Immature respiratory and immune systems may increase infant susceptibility to environmental stressors such as air pollutants^6,7^. According to the World Health Organization (WHO), more than 90% of the global urban population is exposed to air pollution levels exceeding recommended standards^8,9^. In addition to urbanization and traffic-related emissions, broader environmental changes may influence air pollution patterns and respiratory vulnerability. Climate change may further modify pollutant dispersion, atmospheric chemistry, and viral circulation dynamics, potentially affecting exposure profiles and host susceptibility^10,11^.

Particulate matter (PM) and nitrogen oxides have been extensively studied in pediatric respiratory epidemiology, whereas sulfur dioxide (SO_2_) has received comparatively less attention in infant populations^12^. SO_2_ is a soluble pollutant capable of inducing airway irritation, oxidative stress, and inflammatory responses; these mechanisms are compatible with worsening respiratory infections, though not proof of direct causation^13,14^. Current WHO air quality guidelines define exposure limits for SO_2_; however, these standards are based on general population data and do not specifically address infants with ALRI^15,16^.

Epidemiological studies in general and pediatric populations have reported associations between SO_2_ exposure and respiratory morbidity, including pneumonia, ALRIs, and respiratory failure^17-20^. However, most investigations include broad pediatric age ranges and do not specifically focus on infants.

Given the heterogeneity of existing findings and the absence of infant-specific synthesis, this systematic review evaluated whether ambient SO_2_ exposure is associated with increased severity of ALRIs in infants. Following a PECO framework, the review focused on infants (including studies of children up to 5 years when infant-specific estimates were available), ambient SO_2_ exposure compared with lower exposure levels, and severity-related outcomes such as hospitalization, length of stay (LOS), respiratory failure, and mortality.

## MATERIALS AND METHODS

This systematic review followed PRISMA 2020 guidelines^21^. The protocol was registered in OSF (Nº 10.17605/OSF.IO/5TPGC). Studies published between 2015 and 2025 were eligible. The restriction refers to year of publication (not exposure period) and was applied to ensure methodological comparability and contemporary relevance, particularly in light of advances in exposure assessment including higher spatial resolution monitoring and satellite-based estimates, the increasing use of multipollutant models, and improved age-specific analyses in infant populations, as well as to reduce heterogeneity related to earlier analytical approaches. Only studies evaluating severity-related outcomes were included: hospitalization due to ALRI, ICU admission, LOS, need for respiratory support, disease severity scores, or mortality. Eligible clinical conditions included bronchiolitis, pneumonia, and other ALRI. Studies including children aged 0–5 years were eligible if infant-specific results were reported separately. Differences in exposure windows were considered during qualitative synthesis. Eligible study designs included observational studies, cohort studies, time-series analyses, case-crossover studies, and ecological studies. Studies assessing only gestational exposure; systematic reviews; meta-analyses; editorials, commentaries, and letters; and animal or in vitro experimental studies were excluded.

### Information sources and search strategies

A systematic search was conducted in PubMed/MEDLINE, Web of Science (Core Collection), and Embase (Elsevier) to identify studies evaluating the association between atmospheric air pollution, particularly SO_2_, and severity-related outcomes of ALRIs in infants.

### PubMed

The PubMed search strategy combined controlled vocabulary (MeSH terms) and free-text terms. The following search structure was applied: ("Air Pollution"[Mesh] OR "Air Pollutants"[Mesh] OR "SO2"[Mesh] OR "SO2"[tiab] OR "sulphur dioxide"[tiab] OR sulfur dioxide[tiab]) AND ("Respiratory Tract Infections"[Mesh] OR "Bronchiolitis, Viral"[Mesh] OR "Influenza, Human"[Mesh] OR "Respiratory Syncytial Virus Infections"[Mesh] OR "Respiratory Failure"[Mesh] OR "viral respiratory infection*"[tiab] OR "respiratory virus*"[tiab] OR RSV[tiab] OR influenza[tiab] OR bronchiolitis[tiab] OR "hypoxemic acute respiratory failure"[tiab] OR "hypoxaemic acute respiratory failure"[tiab] OR ("acute respiratory failure"[tiab] AND hypoxem*[tiab])) AND ("Infant"[Mesh] OR "Infant, Newborn"[Mesh] OR "Child, Preschool"[Mesh] OR infant*[tiab] OR newborn*[tiab] OR baby[tiab] OR babies[tiab]) AND ("Severity of Illness Index"[Mesh] OR "Hospitalization"[Mesh] OR "Mortality"[Mesh] OR severity[tiab] OR hospitalization[tiab] OR "length of stay"[tiab] OR mortality[tiab]) AND (association*[tiab] OR correlation*[tiab] OR relationship[tiab] OR effect*[tiab] OR exposure[tiab] OR risk[tiab] OR impact[tiab]). Filters were applied for human studies, publication period (2015–2025), and age categories corresponding to Newborn (0–1 month), Infant (0–23 months), and Preschool Child (2–5 years).

### Web of Science (Core Collection)

The search was performed using topic field (TS), combining terms related to air pollution and SO_2_, respiratory infections and respiratory failure, pediatric age groups, severity outcomes, and association measures: ("air pollution" OR "air pollutants" OR "SO2" OR "sulphur dioxide" OR sulfur dioxide) AND ("respiratory tract infection*" OR bronchiolitis OR influenza OR "respiratory syncytial virus" OR RSV OR "respiratory failure" OR "hypoxemic acute respiratory failure" OR "hypoxaemic acute respiratory failure") AND (infant* OR newborn* OR baby OR babies OR "child preschool") AND (severity OR hospitalization OR "length of stay" OR mortality) AND (association* OR correlation* OR relationship OR effect* OR exposure OR risk OR impact). The publication period filter was directly applied (2015–2025).

### Embase (Elsevier)

In Embase, both Emtree controlled vocabulary and free-text terms were used. The search strategy included: (‘air pollution’/exp OR ‘air pollution’ OR ‘air pollutant’/exp OR ‘air pollutant’ OR ‘SO2’/exp OR ‘SO2’ OR ‘sulphur dioxide’/exp OR ‘sulphur dioxide’ OR sulfur dioxide) AND (‘respiratory tract infection’/exp OR ‘respiratory tract infection’ OR ‘bronchiolitis’/exp OR ‘bronchiolitis’ OR ‘influenza’/exp OR ‘influenza’ OR ‘respiratory syncytial virus infection’/exp OR ‘respiratory syncytial virus infection’ OR ‘respiratory failure’/exp OR ‘respiratory failure’ OR ‘hypoxemic acute respiratory failure’/exp OR ‘hypoxemic acute respiratory failure’ OR ‘hypoxaemic acute respiratory failure’/exp OR ‘hypoxaemic acute respiratory failure’) AND (‘infant’/exp OR ‘infant’ OR ‘newborn’/exp OR ‘newborn’ OR infant* OR newborn* OR ‘baby’/exp OR baby OR babies) AND (‘severity of illness’/exp OR ‘severity of illness’ OR ‘hospitalization’/exp OR ‘hospitalization’ OR ‘mortality’/exp OR ‘mortality’ OR ‘length of stay’/exp OR ‘length of stay’ OR ‘severity’/exp OR severity) AND (association* OR correlation* OR ‘relationship’/exp OR relationship OR effect* OR ‘exposure’/exp OR exposure OR ‘risk’/exp OR risk OR ‘impact’/exp OR impact)

The publication period filter was directly applied (2015–2025). The search was restricted to human studies.

A total of 361 records identified across databases were exported to Rayyan^22^ for duplicate removal and two-stage screening (title/abstract followed by full-text review). Initial screening was performed by one reviewer (SEV), with all inclusion and exclusion decisions independently verified by a second reviewer (AAF). Discrepancies, including six records classified as uncertain, were resolved by discussion and consensus.

Data extraction was conducted by one reviewer (SEV) using a standardized form and independently verified by a second reviewer (AAF). Extracted data included study characteristics (title, authors, year, country, design, age range), SO_2_ exposure measures and windows, evaluated outcomes, association estimates, confounder adjustments, and subgroup analyses.

### Quality assessment

Risk of bias in the included studies was assessed using the National Institutes of Health/National Heart, Lung, and Blood Institute (NIH/NHLBI) quality assessment tools, selected according to study design^23^. Cohort studies were evaluated using the NIH Quality Assessment Tool for Observational Cohort and Cross-Sectional Studies. For time-series studies, the NIH Quality Assessment Tool for Before–After (Pre–Post) Studies With No Control Group was applied, with adaptations to reflect characteristics of ecological time-series analyses, including assessment of exposure measurement methods, control for temporal trends and seasonality, lag structure specification, and adjustment for meteorological variables and co-pollutants. Case-crossover studies were evaluated using the NIH Quality Assessment Tool for Case-Control Studies, with items interpreted in light of the case-crossover design, particularly regarding selection of control periods, exposure comparability, and control of time-varying confounders. Ecological studies were assessed using the NIH Quality Assessment Tool for Cross-Sectional Studies, with consideration of aggregated exposure metrics and population-level outcome assessment.

Items not applicable to specific study designs were recorded as such and were not considered in the overall judgment.

Each study was classified as Good, Fair, or Poor according to NIH/NHLBI guidance. Particular emphasis was placed on exposure assessment validity, given its central relevance in environmental epidemiology. Studies using high-resolution or individual-level exposure modeling were eligible for a Good rating when other domains were adequately addressed, whereas studies relying on ecological or fixed-site monitoring exposure metrics were classified as Fair due to the potential for non-differential exposure misclassification. For time-series and case-crossover designs, domains explicitly evaluated included exposure validity, temporality, lag specification, adjustment for meteorological variables and seasonality, and co-pollutant control. Domain-level assessments are summarized in Table 1 to ensure transparency and reproducibility.

**Table 1:** Domain-level methodological assessment and overall quality rating of included studies

Article	Design	Population	Exposure	Outcome	Temporality	Confounding	Co-pollutants	Lag	Main bias	Overall
Belachew *et al*.^24^	Birth cohort	Yes	Yes	Yes	Yes	Yes	Yes	NA	Minimal	Good
Wang *et al*.^32^	Prospective cohort	Yes	Partial	Yes	Yes	Yes	Partial	NA	Temporal granularity	Fair
Nhung *et al*.^30^	Time-series	Yes	Partial	Yes	Yes	Yes	Yes	Yes	Ecological exposure	Fair
Nhung *et al*.^31^	Hospital cohort	Yes	Partial	Yes	Yes	Yes	Yes	Yes	Ecological exposure	Fair
Xu *et al*.^26^	Multi-city TS	Yes	Partial	Yes	Yes	Yes	Yes	Yes	Ecological exposure	Fair
He *et al*.^18^	Time-series	Yes	Partial	Yes	Yes	Yes	Yes	Yes	Ecological exposure	Fair
Zhou *et al*.^19^	Time-series (GAM)	Yes	Partial	Yes	Yes	Yes	Yes	Yes	Ecological inference	Fair
Leung *et al*.^27^	Time-series (DLNM)	Yes	Partial	Yes	Yes	Yes	Yes	Yes	Ecological exposure	Fair
Yitshak-Sade *et al*.^28^	Case-crossover	Yes	Partial	Yes	Yes	Yes	Yes	Yes	Central monitor	Fair
Huang *et al*.^29^	Case-crossover	Yes	Partial	Yes	Yes	Yes	Yes	Yes	Interpolation	Fair
Álvaro-Meca *et al*.^25^	Case-crossover	Yes	Partial	Yes	Yes	Yes	Yes	Yes	Spatial assignment	Fair
Barbosa Neto *et al*.^20^	Population cohort	Yes	Partial	Yes	Yes	Yes	Yes	Yes	Ecological exposure	Fair
Nenna *et al*.^33^	Ecological TS	Yes	Partial	Yes	Yes	Yes	Yes	Yes	Ecological inference	Fair

Yes = Domain fully addressed following NIH/NHLBI criteria; Partial = Addressed with limitations (e.g., ecological exposure assignment, fixed-site monitoring, municipal aggregation, or spatial interpolation); NA = Not applicable to the study design (e.g., lag domain in annual exposure cohort studies); Overall ratings (Good/Fair) were determined using a strict and conservative uniform strategy, with emphasis on exposure assessment validity.

### Certainty of the evidence

In addition to the study-level risk of bias assessment, the overall certainty of the evidence across outcomes was evaluated using an adapted GRADE approach. This assessment considered risk of bias, inconsistency, indirectness, imprecision, and publication bias. Given the heterogeneity in study designs, exposure metrics, and outcome definitions, the assessment was conducted qualitatively without quantitative pooling. The overall certainty was classified as high, moderate, low, or very low for each outcome.

### Data synthesis

A descriptive narrative synthesis was performed, structured by type of outcome and by the magnitude and direction of associations. The data synthesis was conducted by authors SEV and AAF. When studies reported multiple lag structures, the lag identified by the authors as the primary exposure window or emphasized in the main results was considered in the qualitative synthesis. Additional lag estimates were described when clinically or methodologically relevant. When multiple exposure increments were reported (e.g., per 1 µg/m^3^, per 10 µg/m^3^, per interquartile range, or percentile-based comparisons), the increment specified in the primary model of the study was retained. When standardized increments (e.g., per 10 µg/m^3^) were available, these were preferentially described to facilitate comparability across studies. When both single-pollutant and multipollutant models were presented, estimates from the most fully adjusted model were prioritized. Subgroup analyses (e.g., by age or sex) were reported when relevant to infant populations. A consistent hierarchical approach was applied during data extraction to ensure transparency and minimize selective reporting bias.

Heterogeneity across studies (designs, exposure windows, measurement units, and SO_2_ concentrations) precluded conducting a meta-analysis.

## RESULTS

**Figure 1:**
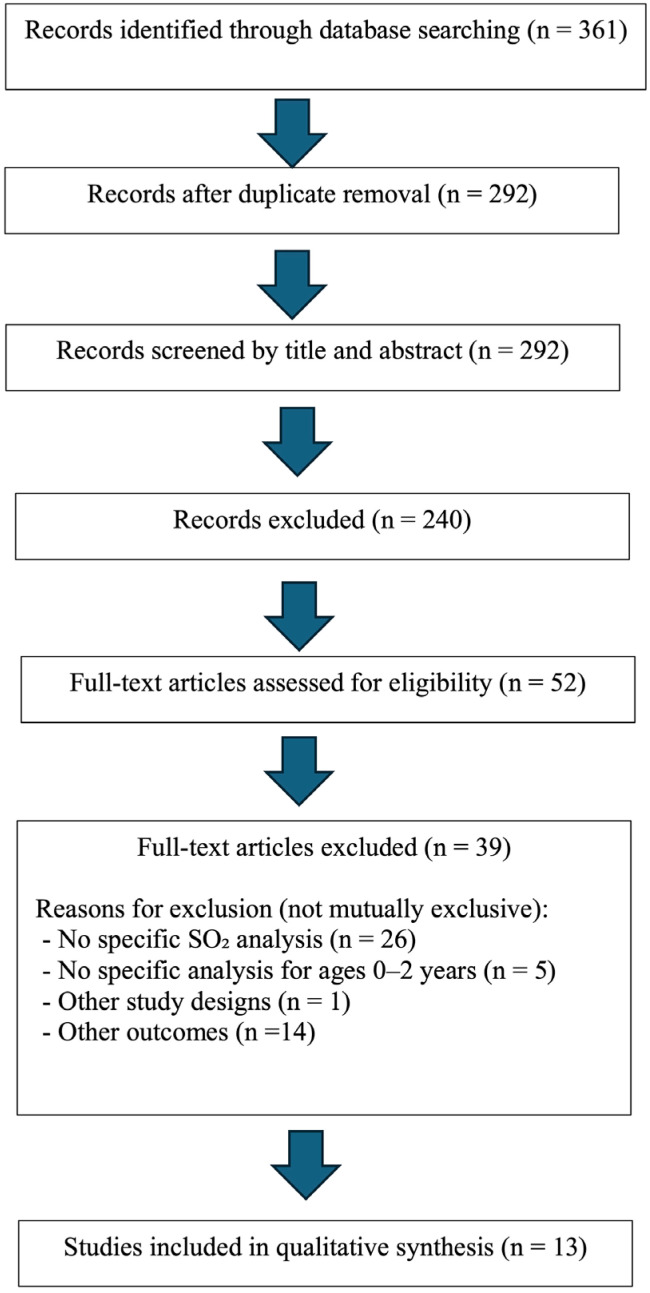
PRISMA 2020 flow diagram of the study selection process.

A total of 361 records were identified via database searching. After removal of duplicates, 292 records remained and were screened by title and abstract. Of these, 240 were excluded based on predefined eligibility criteria. Fifty-two full-text articles were assessed for eligibility. Of these, 39 were excluded after full-text review. The primary reasons for exclusion were absence of specific SO_2_ analysis (n = 26), lack of age-specific estimates for children aged 0–2 years (n = 5), outcomes not related to disease severity (n = 14), and non-eligible study design (n = 1). Some studies met more than one exclusion criterion; therefore, counts are not mutually exclusive. Thirteen studies met all inclusion criteria and were included in the qualitative synthesis (Figure 1).

Among the thirteen included studies, four were classified as Good and nine as Fair quality according to NIH/NHLBI tools (Table 1). Domain-level assessment demonstrated that population definition, outcome ascertainment, temporality, lag specification, and adjustment for meteorological variables and seasonality were adequately addressed across studies. However, exposure validity was classified as partially addressed in nine studies due to reliance on ecological exposure assignment (e.g., fixed-site monitoring, municipal-level aggregation, or spatial interpolation), reflecting the inherent risk of non-differential exposure misclassification. This methodological limitation may attenuate effect estimates and therefore informed the Fair classification. Studies rated as Good incorporated higher-resolution or individual-level exposure modeling and fulfilled all major NIH/NHLBI domains without exposure-related limitations warranting partial classification.

A total of thirteen studies published between 2017 and 2024 were conducted in Israel, China, Hong Kong, Italy, Vietnam, Finland, Brazil, and Spain. Study designs included time-series, case-crossover, cohort studies, and population-based observational analyses. Exposure assessment methods varied in spatial resolution and modeling approach.

Reported mean ambient SO_2_ concentrations varied substantially across studies, ranging from 0.9–1 µg/m^3^ in Italy and 4.8 µg/m^3^ in Israel to approximately 10–12 µg/m^3^ in Finland and Brazil and 12–14 µg/m^3^ in one Chinese cohort. Higher mean concentrations were reported in other studies from China and Vietnam (28.7–39 µg/m^3^). One Spanish study did not report concentration values. Overall, the included studies covered a broad range of ambient SO_2_ exposure levels across diverse settings.

Eight studies reported statistically significant positive associations between short-term SO_2_ exposure and increased risk or severity of respiratory outcomes in infants; one study observed a positive but non-significant trend, and four reported no statistically significant associations. Reported severity outcomes included hospitalization for pneumonia or bronchiolitis, acute lower respiratory infections requiring hospital care, length of stay, and mortality. Cross-study patterns are summarized in Table 2.

**Table 2:** Cross-study methodological patterns and consistency of findings

Article	Design	Primary exposure window	Exposure increment	Adjustment model	Outcome	Direction of association	Statistically significant
Belachew *et al*.^24^	Birth cohort	Annual	Continuous / mixture	WQS model	Severe LRTI	Positive association (IRR 2.81)	Yes
Wang *et al*.^32^	Prospective cohort	Annual	Continuous	Random Forest (multivariable)	Pneumonia severity	Positive association	Yes
Nhung *et al*.^30^	Time-series	Lag0–6	Per IQR	Multipollutant	Pneumonia / Bronchitis	Small positive association (non-significant)	No
Nhung *et al*.^31^	Hospital cohort	Lag1–4	Per IQR	Multipollutant	Length of stay	Inverse association (shorter LOS)	Yes
Xu *et al*.^26^	Multi-city time-series	Lag01	Per 10 µg/m^3^	Multipollutant	Respiratory hospitalization	Positive association (0.60% increase)	Yes
He *et al*.^18^	Time-series	Lag010 cumulative	Per 10 µg/m^3^	Multipollutant	Pneumonia / Bronchitis	Positive association	Yes
Zhou *et al*.^19^	Time-series (GAM)	Lag03	Per 10 µg/m^3^	Multipollutant GAM	Hospitalization	Positive association (11.21% increase)	Yes
Leung *et al*.^27^	Time-series (DLNM)	Distributed lag	Percentile-based	Multipollutant DLNM	Bronchiolitis hospitalization	Positive trend (non-significant)	No
Yitshak-Sade *et al*.^28^	Case-crossover	Lag0–7	Per IQR (3.14 µg/m^3^)	Multipollutant	Bronchiolitis hospitalization	Null	No
Huang *et al*.^29^	Case-crossover	Lag4	Per 10 µg/m^3^	Multipollutant	Pneumonia	Positive association	Yes
Álvaro-Meca *et al*.^25^	Case-crossover	1–2 week windows	Continuous	Conditional logistic	Viral ALRI hospitalization	Positive association	Yes
Barbosa Neto *et al*.^20^	Population-based	30–60 day mean	Continuous	Multipollutant regression	LOS & mortality	Positive association	Yes
Nenna *et al*.^33^	Time-series	Daily lags	Per unit	Multipollutant	RSV bronchiolitis	Null	No

Primary exposure window = main lag or averaging period; Exposure increment = metric used to express effect estimates; Positive association = statistically significant increase; Positive trend (NS) = positive estimate not statistically significant; Small positive association (NS) = modest positive estimate not statistically significant; Null = no statistically significant association (95% CI includes null value).

Most included studies evaluated short-term exposure using daily lag structures or moving averages preceding the outcome, whereas one birth cohort study assessed annual exposure during the first year of life.

### Quantitative synthesis

Given variability in exposure metrics (per 1 µg/m^3^, per 10 µg/m^3^, per IQR, or percentile-based comparisons), lag structures, modeling strategies, and outcome definitions, quantitative pooling was not performed.

Statistically significant associations were more frequently observed in time-series and case-crossover designs evaluating short-term cumulative exposure windows (lag01–lag03 and 1–2-week averages). Effect estimates per 10 µg/m^3^ increase were generally modest but statistically significant in specific lag structures. Cohort-based and mixture-model analyses also identified associations with severity-related outcomes, including hospitalization, progression to severe ALRI, longer LOS, and mortality.

Null findings were frequently observed in low-exposure settings or in analyses relying on ecological exposure metrics without refined spatial modeling. Overall, effect magnitude varied across regions and exposure distributions, with greater consistency observed in short-term exposure analyses conducted in urban contexts with higher SO_2_ variability.

The certainty of the evidence across outcomes was assessed using an adapted GRADE approach (Table 3). All included studies contributed to at least one outcome, and several contributed to more than one outcome. For hospitalization-related outcomes, contributing studies included He *et al*.^18^, Zhou *et al*.^19^, Belachew *et al*.^24^, Álvaro-Meca *et al*.^25^, Xu *et al*.^26^, Leung *et al*.^27^, Yitshak-Sade *et al*.^28^, Huang *et al*.^29^, and Nhung *et al*.^30^. For length of stay, contributing studies included Barbosa Neto *et al*.^20^ and Nhung *et al*.^31^. For progression to severe disease, studies included Belachew *et al*.^24^ and Wang *et al*.^32^ Mortality outcomes were primarily evaluated in the study by Barbosa Neto *et al*.^20^ Overall, the certainty was judged as low to very low. This reflects the observational nature of the included studies, heterogeneity in exposure assessment, lag structures, and outcome definitions, as well as potential residual confounding. Certainty was relatively higher for hospitalization-related outcomes, although still limited, and lower for outcomes such as mortality and progression to severe disease due to the small number of studies and imprecision.

**Table 3:** Certainty of the evidence according to an adapted GRADE approach

Outcome	No. of studies	Study design	Risk of bias	Inconsistency	Indirectness	Imprecision	Publication bias	Certainty
Hospitalization for ALRI	9	Observational (time-series, case-crossover, cohort)	Moderate (ecological exposure in several studies)	Moderate (heterogeneity in lag structures and exposure metrics)	Low	Moderate	Not assessable	Low
Length of stay (LOS)	4	Observational	Moderate	Moderate	Low	Moderate (limited number of studies)	Not assessable	Low
Progression to severe disease	3	Observational	Moderate	Moderate	Low	Serious (few studies)	Not assessable	Very low
Mortality	2	Observational	Moderate	Serious (very limited evidence)	Low	Serious (few events/studies)	Not assessable	Very low

Certainty of the evidence was assessed using an adapted qualitative GRADE approach. ‘No. of studies’ represents the maximum number of studies contributing to each outcome. Risk of bias reflects study-level limitations identified using NIH/NHLBI tools. Inconsistency refers to variability in effect estimates across studies. Indirectness considers applicability to infants and outcomes of interest. Imprecision reflects limited number of studies and variability in estimates. Publication bias was not formally assessed due to small number of studies and heterogeneity.

## DISCUSSION

This systematic review suggests that short-term atmospheric SO_2_ exposure in early life is associated with increased severity of respiratory infections in infants, particularly hospitalization, LOS, and progression to severe disease. Across the 13 included studies, eight reported statistically significant positive associations, four did not observe significant associations, and one identified an inverse association for LOS in the primary analysis. This inverse finding was attenuated in subgroup and multipollutant models, suggesting limited stability. The magnitude of reported effects varied according to exposure increment and lag structure. This variability likely reflects substantial methodological heterogeneity across studies, including differences in exposure windows (annual averages versus short-term daily lags and cumulative structures), exposure metrics (per 1 µg/m^3^, per 10 µg/m^3^, or per interquartile range), outcome definitions, and analytical strategies such as multipollutant adjustment and alternative modeling approaches. These considerations are consistent with the overall certainty of the evidence, which was judged as low to very low using an adapted GRADE approach. This reflects not only the observational design of the included studies but also heterogeneity in exposure assessment, variability in analytical approaches, and potential residual confounding across settings.

Within this clinical framework, hospitalization for ALRI constitutes a meaningful marker of disease severity. In the study by Belachew *et al*.^24^, the mean first-year SO_2_ concentration was 10.43 µg/m^3^ (SD 4.76; 75th percentile 12.59 µg/m^3^; maximum 43.16 µg/m^3^). Infants in the highest exposure quartile had a significantly higher rate of hospital-diagnosed ALRIs compared with those in the lowest quartile (IRR 2.81, 95% CI 1.35–5.83). SO_2_ was the largest contributor to the multipollutant mixture index. Notably, this study used high-resolution temporal and spatial exposure modeling and a weighted quantile sum approach to account for pollutant mixtures.

A similar severity indicator was used in the Spanish study by Álvaro-Meca *et al*.^25^, conducted with more than 30,000 infants with ALRI, in which SO_2_ exposure on the day of admission showed a significant association with hospitalization. Although the exposure method was based on fixed monitoring stations assigned to postal codes, without fine spatial modeling, the wide spatial coverage and large number of cases provide robustness to the finding. The study population consisted predominantly of infants with bronchiolitis, and more than half of the cases were infections caused by RSV, reinforcing that the results mainly reflect associations between pollution exposure and typical RSV-related severe conditions in early childhood.

Similarly, hospital-based analyses in other urban settings have reported comparable patterns. In Wuhan, short-term SO_2_ exposure was associated with pneumonia hospitalization at specific lags, particularly during colder periods and among males. However, SO_2_ exposure was estimated using inverse distance weighting (R^2^ = 0.65), a method with lower spatial resolution than land-use regression models used for other pollutants, which may have attenuated effect estimates^29^.

Large-scale time-series studies conducted in different Chinese regions show a broadly consistent pattern of association between short-term SO_2_ exposure and severity-related respiratory outcomes. In a multi-city analysis including nearly 900,000 hospitalizations, Xu *et al*.^26^ observed a statistically significant increase in respiratory admissions per 10 µg/m^3^ increment, with stronger effects among children under one year of age. Similarly, He *et al*.^18^ reported consistent associations even with small per-unit increments (1 µg/m^3^), particularly at cumulative lags. Zhou *et al*.^19^, using generalized additive models, identified a substantial increase in hospitalization risk at short cumulative lags. In contrast, Leung *et al*.^27^, applying distributed lag non-linear models in Hong Kong, observed a positive but non-significant association, highlighting the influence of exposure distribution and local pollutant mixtures on effect magnitude.

Beyond hospitalization risk, some investigations assessed in-hospital severity progression. In the study by Barbosa Neto *et al*.^20^, conducted in Sao Paulo, the mean daily SO_2_ concentration was 3.7 µg/m^3^ (SD 2.4; IQR 2.7 µg/m^3^). An increase of one IQR in SO_2_ (2.7 µg/m^3^) was associated with a 6.8% increase in LOS (95% CI 0.3%–13.7%) and with higher in-hospital mortality (OR 1.68, 95% CI 1.03–2.75). Associations were observed for acute and subchronic exposure windows and were amplified on hot and dry days. These findings were derived from infants hospitalized with acute respiratory failure, a condition frequently triggered by viral respiratory infections. In the study by Zhi-Bo Wang *et al*.^32^, conducted in Chongqing, SO_2_ was part of the group of pollutants associated with the likelihood of progression to severe pneumonia, contributing significantly to the predictive model of severity.

The positive associations between SO_2_ exposure and severity outcomes contrast with studies conducted in Hanoi, Vietnam. The time-series study examining daily risk of ALRI admissions found no effect of SO_2_ on increased hospitalizations among infants^31^. Another study by the same group found no consistent associations between SO_2_ exposure and LOS for respiratory infections^26^. These findings suggest that, in the specific context of Hanoi, SO_2_ was not the main marker of respiratory toxicity in infants, unlike NO^2^ and particulate matter, which showed consistent effects^30,31^.

Null or inverse associations should be interpreted cautiously, as they may reflect methodological limitations rather than a true absence of pollutant impact. The study by Yitshak-Sade *et al*.^28^ did not find associations between daily mean SO_2_ and bronchiolitis hospitalizations in Israel. Unlike PM, which had its concentration modeled with high spatial resolution using satellite data and environmental variables, SO_2_ concentration was obtained exclusively from a single central monitor, without accounting for intra-urban variation or the actual locations of residences, potentially leading to non-differential exposure misclassification and attenuation of effect estimates^28^.

Comparison among these studies also shows that the impact of SO_2_ on severity appears more evident in urban scenarios where pollutant mixtures are dominated by vehicular or stationary combustion, such as Sao Paulo and Finland, but less evident in regions where particles and NO^2^ play a predominant role, such as Hanoi^20,24,30,31^.

While epidemiological associations do not establish causality, several biological mechanisms have been described that are compatible with the observed patterns. These mechanisms provide biological plausibility but should not be interpreted as direct confirmation of the epidemiological findings. After inhalation, SO_2_ is rapidly converted into sulfite and bisulfite, which are reactive metabolites capable of generating reactive oxygen species, inducing oxidative stress, and triggering inflammation, causing epithelial damage, increased permeability, release of pro-inflammatory cytokines, and neutrophil recruitment^13-15^. SO_2_ also impairs host defense against inhaled viruses by compromising epithelial barrier integrity and ciliary function^33-35^. In infants with viral infection, this combination of epithelial damage, more viscous mucus, and reduced clearance facilitates higher viral load in small airways, promotes mucus plugging, and worsens gas exchange, which may contribute to increased LOS and mortality, consistent with the epidemiological associations reported in studies such as Barbosa Neto *et al*.^20^. Although these mechanisms are biologically plausible and supported by experimental evidence, the observational nature of the included studies does not allow definitive causal inference.

SO_2_ also modulates innate and antiviral immunity by altering interferon production, macrophage and dendritic cell function, and the balance between Th1 and Th2 responses. Experimental models suggest that urban pollution amplifies virus-induced airway inflammation and delays viral clearance^36-39^. In infants, such dysregulation may contribute to exaggerated inflammatory responses and more severe respiratory outcomes^20,24^.

The greater vulnerability of infants to SO_2_ also results from physiological characteristics of this age group, such as higher respiratory rate, greater inhaled volume per body weight, smaller airway caliber, and pulmonary and immunological immaturity. Lower antioxidant and detoxification capacity and greater pollutant deposition in small airways amplify cytotoxic and inflammatory effects of SO_2_ metabolites^40^. In addition, pulmonary microRNAs, which regulate inflammation, antiviral responses, and epithelial integrity, are undergoing rapid developmental changes early in life^41,42^. This developmental instability may reduce regulatory control of inflammatory and epithelial repair responses following environmental insults^40^. These factors, combined with innate immune limitations in neonates, such as lower production of regulatory cytokines, reduced antigen-presenting cell activity, and less efficient antiviral responses, intensify SO_2_ effects in this age group^5^.

Finally, SO_2_ rarely acts in isolation and is commonly present in mixtures with NO^2^, CO, and PM, contributing to secondary sulfate formation in fine and ultrafine PM^43^. In some contexts, SO_2_ may function primarily as an indicator of broader combustion-related mixtures, and observed associations may reflect combined pollutant effects. Similar challenges in disentangling independent effects arise for other traffic- and combustion-related pollutants due to shared sources and strong intercorrelations; therefore, epidemiological associations should be interpreted within the context of complex atmospheric mixtures rather than as strictly independent effects in all settings. In scenarios where SO_2_ appears as a strong risk marker (Belachew *et al*.^24^, Barbosa Neto *et al*.^20^, and Álvaro-Meca *et al*.^25^), it may reflect both its own biological activity and correlation with combustion-related mixtures. Conversely, in settings such as Hanoi and Rome, other pollutants, mainly NO^2^ and PM, appeared more strongly associated with severity outcomes.

Among the included studies, approaches to co-pollutant adjustment were heterogeneous. Some investigations relied on single-pollutant models, while others incorporated multipollutant frameworks including PM_2.5_, PM^10^, and NO^2^. In several settings (Wuhan and Vietnam), SO_2_ associations attenuated after adjustment for PM or NO^2^, suggesting shared source contributions. These differences highlight the methodological challenges of isolating independent pollutant effects in observational air pollution research.

Additionally, co-exposure to SO_2_ and fine PM is biologically compatible with enhanced oxidative stress and airway inflammation within multipollutant environments. This is biologically plausible given the established role of SO_2_ as a precursor of secondary sulfate formation, which contributes to the mass and physicochemical properties of fine PM in combustion-related atmospheres^43^. Accordingly, observed associations in multipollutant models may reflect not only statistical collinearity but also the integrated effects of chemically related pollutants acting within complex atmospheric mixtures.

The associations observed for SO_2_ should be interpreted within the context of pollutant mixtures. In some settings, such as Wuhan and Vietnam, SO_2_ effects attenuated after adjustment for PM^2.5^, PM^10^, or NO^2^, suggesting shared combustion sources and collinearity. In contrast, in studies such as those by Belachew *et al*.^24^ and Barbosa Neto *et al*.^20^, SO_2_ remained associated with severity-related outcomes in multipollutant models, and in the Finnish cohort it contributed substantially to the mixture index. These patterns are consistent with the possibility that SO_2_ may act both as an indicator of broader combustion-related pollution and, in certain contexts, as a contributor within complex atmospheric mixtures. Given the observational design and intercorrelation among pollutants, these findings support a mixture-related interpretation rather than definitive evidence of independent or synergistic effects.

Although some studies propose a possible protective effect of SO_2_, suggesting that acidic environments might reduce viral survival and transmission, these findings are weak, mostly ecological, based on general populations, and influenced by climatic confounders^44,45^. Time-series analyses often reflect meteorological patterns affecting viral seasonality, making it difficult to separate pollution effects from climate influences. Laboratory experiments suggesting antiviral effects of SO_2_ were conducted under artificial conditions without direct relevance to human exposure^46^.

These findings have implications for public health policies. Air quality standards based on population averages may fail to protect vulnerable groups such as infants. In several included studies, statistically significant associations were observed within exposure ranges below the current WHO 24-hour guideline (40 µg/m^3^)^15^. However, most studies evaluated short-term exposure increments rather than specific exposure thresholds, and therefore do not establish a safe or unsafe concentration level. These findings highlight the need for further research focusing on vulnerable populations such as infants.

Measures specifically targeting infant health may be needed. Policies for vehicle fleet renewal, low-emission zones, improved monitoring near childcare centers and health services, enhanced filtration in indoor environments, guidance for families on pollution peaks, and urban planning that reduces heavy-vehicle traffic and expands green areas may help mitigate SO_2_ effects.

In addition to classical gaseous and particulate pollutants, emerging evidence suggests that air pollution–related health effects may be influenced by broader environmental dynamics and complex exposure patterns. Recent studies have highlighted substantial variability in gaseous pollutant concentrations and associated health risks across different urban contexts, including during periods of abrupt emission changes such as the COVID-19 pandemic, as well as in rapidly urbanizing regions with heterogeneous pollution profiles^47-50^. Although these aspects were not the primary focus of the present review, which specifically addressed SO_2_ exposure and severity-related respiratory outcomes in infants, they reinforce the concept that pollution-related morbidity is context-dependent and may reflect interactions within complex atmospheric mixtures. These considerations support interpreting SO_2_ not only as an isolated pollutant but also as a component of broader environmental exposure profiles.

### Limitations of the study

The main limitations of this review include heterogeneity in study designs and exposure assessment methods, which reduce comparability. Exposure based on aggregated means may have introduced non-differential misclassification, while collinearity among pollutants may have contributed to residual confounding. Limited variability in SO_2_ levels in certain regions also restricted the ability to detect associations. Additionally, null findings in some settings may reflect true contextual differences in pollutant mixtures and dominant emission sources rather than solely methodological limitations. Standardized methods and improved exposure characterization are needed. Importantly, most of the included studies were observational and subject to residual confounding, including meteorological factors, viral seasonality, socioeconomic status, healthcare access, and co-pollutant correlations. Differences in statistical modeling strategies (e.g., lag structure, single vs. multipollutant models, exposure metrics) may also explain variability in effect estimates. Therefore, the observed associations should be interpreted as evidence of potential contribution rather than definitive causal attribution. As this review synthesized heterogeneous observational studies without producing pooled quantitative estimates, formal assessment of publication bias (e.g., funnel plots or Egger's regression test) was not performed. The small number of included studies (n = 13) and substantial heterogeneity in study design, exposure metrics, lag structures, and outcome definitions precluded meaningful application of these methods. Nevertheless, publication bias cannot be excluded.

This systematic review identified evidence suggesting that short-term exposure to SO_2_ may be associated with increased severity of respiratory infections in infants, including higher risk of hospitalization, longer LOS, and progression to severe disease in certain settings. However, findings were heterogeneous across regions, and effect estimates varied according to exposure assessment methods and multipollutant modeling approaches.

Given the observational design of the included studies and the challenges of disentangling independent pollutant effects within complex atmospheric mixtures, the evidence does not allow definitive causal inference.

These findings have potential public health relevance, particularly for infants as a physiologically vulnerable population. Although the evidence does not support specific regulatory thresholds or definitive causal claims, it is compatible with precautionary efforts to reduce infant exposure to combustion-related urban air pollution^15,16^. Further high-quality epidemiological research is needed to clarify independent pollutant effects and inform proportionate policy decisions.

## CONCLUSION

This review suggests that short-term atmospheric SO_2_ exposure may be associated with increased severity of respiratory infections in infants, particularly hospitalization in some urban settings. The most consistent associations were observed for short-term exposure and hospitalization-related outcomes, especially in time-series and case-crossover studies conducted in urban environments with higher pollutant variability. However, given the observational and heterogeneous nature of the evidence, these results should be interpreted cautiously. Further high-quality studies with standardized exposure assessment and multipollutant modeling are needed to clarify independent effects and inform public health policy.

From a precautionary perspective, these findings support efforts to reduce infant exposure to combustion-related air pollution, including strengthening air quality monitoring in areas frequented by infants and considering whether current air quality guidelines, including WHO recommendations, adequately protect vulnerable populations.

## Data Availability

The complete anonymized dataset supporting the findings of this study is included within the article itself.
